# Prolyl oligopeptidase is inhibited in relapsing-remitting multiple sclerosis

**DOI:** 10.1186/1742-2094-7-23

**Published:** 2010-04-06

**Authors:** Jofre Tenorio-Laranga, Francisco Coret-Ferrer, Buenaventura Casanova-Estruch, María Burgal, J Arturo García-Horsman

**Affiliations:** 1 Department of Neurobiology, Centro de Investigación Príncipe Felipe, Valencia, Spain; 2 Division of Pharmacology and Toxicology, University of Helsinki, Finland; 3 Hospital Clínico Universitario de Valencia, Valencia, Spain; 4 Hospital Universitario La Fe, Valencia, Spain

## Abstract

**Background:**

Multiple sclerosis (MS) is a complex, inflammatory and neurodegenerative disease of the central nervous system leading to long-term disability. Recent studies indicate a close association between inflammation and neurodegeneration in all lesions and disease stages of MS. Prolyl oligopeptidase (POP) is a proline-specific serine protease that cleaves several neuroactive peptides. This peptidase has been implicated in neurodegeneration, as well as in the modulation of the inflammatory response.

**Methods:**

We examined plasma POP and the levels of an endogenous POP inhibitor from relapsing remitting MS patients and compared these with healthy controls, by monitoring the fluorescent changes due to standard fluorescently labelled substrate cleavage. We analysed the data in relationship to patient age and disease disability status.

**Results:**

We observed a significant decrease in POP activity in plasma of relapsing remitting MS patients relative to healthy controls, coupled with an increase of POP endogenous inhibitor. The POP activity was also correlated with patient age and disability status. The lowered POP activity from plasma of MS patients could be rescued by reductants

**Conclusions:**

The decrease in circulating POP activity measured in MS is reverted by reductants. This suggests that POP inactivation in MS might be a result of the oxidative conditions prevailing in the plasma of the diseased patients. Plasma levels of POP activity as well as those of their endogenous inhibitor are suggested as biomarkers of inflammation and oxidative stress in MS.

## Background

Prolyl oligopeptidase (POP) is a proline-specific endopeptidase, highly expressed in brain, that cleaves neuroactive peptides implicated in memory, learning and also in neurodegeneration [1,2]. The distribution of the enzyme across the brain indicates that POP may be involved in the thalamocortical neurotransmission, memory and learning functions of hippocampal formation and GABAergic regulation of voluntary movements [3-5]. POP inhibitors are neuroprotective in certain experimental settings, for instance in brain ischemia and in T-cell activation-induced cell death [1]. Active POP increases the aggregation rate of α-synuclein in vitro [6], and serum levels of this activity are lower in different stages of depression and higher in mania [7]. Furthermore, the effect of the mood-stabilizing drugs, lithium, carbamazepine and valproic acid, can be reversed by POP inhibition [8]. Additionally, POP inhibitors can reverse memory loss in rodents under several experimental conditions, such as scopolamine administration [9] or brain ischemia [10]. A POP endogenous inhibitor has been described [11] and proposed as a regulator of POP activity in cells, tissues and fluids [1]. Moreover, myelin basic protein has been suggested as a substrate for POP [12].

Recent studies indicate a close association between inflammation and neurodegeneration in all lesions and disease stages of MS [13]. MS is associated to immunological changes elicited by endogenous myelin-associated antigens such as myelin oligodendrocyte glycoprotein, proteolipoprotein, and myelin basic protein [14]. Acute lesions are thought to result when activated T cells, responsive to these and other potential antigens, traffic to the central nervous system and trigger a cascade of inflammatory events. Widespread axonal degeneration and brain atrophy appear early in the disease course and are prominent in progressive forms of MS. During an acute inflammatory attack, increases in free radicals lead to mitochondrial damage and decreased ATP production, which would be expected to inhibit axonal transport, including the transport of mitochondria [15]. Inflammation also activates signalling pathways that inhibit axonal transport.

Recently a direct role for POP as a modulator of the inflammatory response has been proposed [16]. This response involves a multistep process during matrix metalloproteases (MMPs) mediated collagen degradation, resulting in formation of the peptide, N-acetylated-proline-glycine-proline (N-α-PGP), a POP product. This peptide has been described as a neutrophil chemoattractant and also as a stimulator of superoxide production [17]. In addition, PGP is a biomarker for chronic obstructive pulmonary disease (COPD), an inflammatory disorder [18]. This study has also shown that POP, in conjunction with MMPs, is required for production of PGP. Kamori and collaborators [19], have showed that POP activity levels are increased in knee joint synovial membrane of patients with rheumatoid arthritis, a systemic inflammatory disorder.

On the other hand, recent studies have shown that POP is a mediator of the toxic effect that reactive microglia cells have in a neural cell culture model. This effect is reduced by POP inhibitors [20]. The presence of reactive microglia cells has been observed in Alzheimer's, Parkinson's, Pick's and Huntington's diseases, amyotrophic lateral sclerosis, AIDS encephalopathy, and MS [21]. Additionally, microglia are the principal cells that mediate innate immune responses in the CNS.

POP has been found to be responsible for the generation of the thymosin β-4 derived peptide N-acetyl-seryl-aspartyl-lysyl-proline (Ac-SDKP, [22]). Ac-SDKP is involved in inflammatory cell infiltration, perivascular fibrosis and glomerulosclerosis [23], and in human malignant tumours [24]. We have evidence that β-thymosin is indeed a physiological substrate of POP in brain [25].

Alterations in plasma POP activity have been found in several neurological diseases, but to our knowledge, the possible involvement of this peptidase in MS has never been studied. POP has implications in several processes relevant to MS: 1) it has a role in the inflammatory response through generation of key peptides from degradation of extracellular matrix proteins; 2) it is related to microglia activation and T cell response; 3) it is involved in degradation of myelin basic protein; and 4) it has a role in axonal transport. Based in all these clues, we hypothesised that POP activity would be altered in MS. In this study, we have analysed the levels of POP activity, and those of an endogenous POP inhibitor, in a Spanish sample of clinically diagnosed MS patients. We also analysed the results in relation to patient age and disability in search of possible correlations.

## Methods

### Patients

Eleven patients, 7 females and 4 males, of an average age of 32 ± 7 years were used in this study. All the patients had confirmed clinical diagnosis of MS according to the modified McDonald criteria [26]. Furthermore, all the subjects were clinically classified as having the relapsing remitting form of MS according to the criteria described by Lublin and Reingold [27]. The time of evolution of the disease from the onset of the dysfunction was between 2 to 156 months (mean 71 ± 60 months), and the Kurtzke Expanded Disability Status Scale (EDSS) [28] score of the MS patients ranged from 1.5 to 3.5 at the time when the samples were taken. All patients were on interferon-β therapy.

### Controls

Age-matched healthy volunteers (8 females and 9 males) were recruited for this study. None of the controls had a history of neurological symptoms or of any chronic disease.

### Blood sampling

Blood was collected from controls and patients by venipuncture from an antecubital vein. Blood (8.5 ml/tube) was directly drawn into two evacuated tubes containing anticoagulants (buffered citrate, EDTA). After collection, blood specimens were spun at 10,000 × g for 1 minute at 4°C. Supernatant plasma was aliquoted and stored at -80°C.

### Ethical considerations

This study was approved by the Ethical Committee of the Centro de Investigación Principe Felipe, Valencia, Spain and followed local and European regulations. All subjects gave their informed consent for the blood collection.

### Partial purification of POP

Prolyl oligopeptidase was purified from porcine brain by ion exchange chromatography as described before [29].

### Enzymatic assay

**Figure 1:**
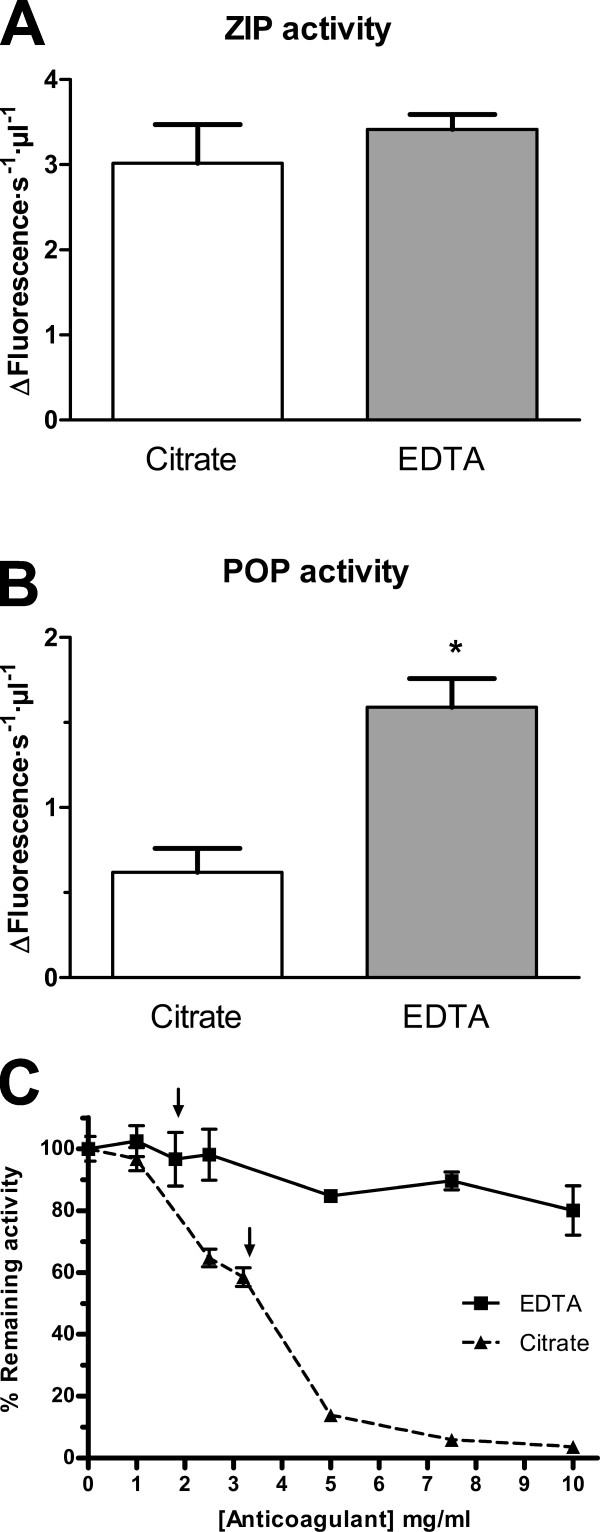
**Prolyl oligopeptidase activity is sensitive to chelating agents but the alternative ZIP activity is not**. Prolyl endopeptidase activity in plasma from healthy controls collected in the presence of citrate (gray bars) or EDTA (white bars). **A**. Z-Pro-Prolinal insensitive prolyl endopeptidase (ZIP). **B**. POP activity, *, *p*-value = 0.037. n = 4. **C**. Effect of increasing concentrations of citrate (-▲-) or EDTA (-■-) on human recombinant purified POP activity. Concentrations of 1.8 mg/ml EDTA or 3.2 mg/ml citrate (arrows) were used in the experiments shown in **A**. and **B**.

As repeated cycles of freeze-thawing inactivates POP [30], all the samples were analysed after only one freezing cycle. Plasma aliquots (25 μl) were added to each well of a 96-well plate which contained 220 μl 100 mM of sodium phosphate pH 7.0 and pre-incubated 30 min at 30°C. Then, 5 μl of the substrate Z-Gly-Pro-amino methyl coumarin (Z-GP-AMC, 10 mM, Bachem, Wheil am Rhein, Germany) were added and the activity was assayed by measuring the increase of fluorescence released from amino methyl coumarin (AMC). The measures were taken every minute over a time course of 90 min at 30°C. Two enzymes account for the total prolyl endopeptidase detected in plasma [31]. One is resistant to specific POP inhibitors and is referred to as Z-Pro-Prolinal (ZPP)-insensitive prolyl endopeptidase, or ZIP activity. A second, POP activity, corresponds to the fraction sensitive to the inhibitor. Accordingly, we report POP activity as the difference between the total activity and the fraction resistant to 50 nM of ZPP (Fig. 1).

### POP endogenous inhibitor

For determination of POP endogenous inhibitor in plasma, purified porcine POP (800 ng) was used in the fluorescence activity assay described above, with or without pre-incubation with 25 μl of plasma. Endogenous POP inhibitor level is reported as the percentage of inhibition caused by the plasma, in these conditions, on purified POP activity. The plasma prolyl endopeptidase activity contribution was less than 10%, and was subtracted in all cases. All assays were done by triplicate.

### Data analysis

All analysis was performed using GraphPad Prism 4 software (La Jolla, CA, USA). For comparison between different groups, student's unpaired *t *test and one-way ANOVA, with Tukey post hoc test, analysis were used. Differences were considered statistically significant when a p value was <0.05. Results are expressed as means ± S.E.M.

## Results

### Plasma collection conditions affect prolyl endopeptidase activity and stability

Important variations in activity and stability of POP due to preparation and assay conditions have been noticed before [30]. On the other hand, two different enzymes in plasma, with prolyl endopeptidase activity, have been described [31]. Apart from classical POP, the alternative activity is insensitive to POP-specific inhibitors, and is called Z-Pro-Prolinal insensitive peptidase or ZIP. This activity has been attributed to the action of fibrinogen activated protein α (FAPα) or seprase [32]. On the other hand, Breen and collaborators [31] have reported that cation chelators, which are currently used during plasma preparation, affect POP activity. Our experiments indicate that while neither citrate nor EDTA in the plasma affected the levels of ZIP activity (Fig. 1A), activity of POP, or the Z-pro-prolinal sensitive activity level, was found to be substantially lower in the presence of citrate (p < 0.05). EDTA-containing plasma seems to have higher levels of POP (Fig. 1B). To verify the sensitivity to these chelators, we directly assayed their effect on the activity of purified POP (Fig. 1C). We observed that both compounds inhibit POP activity. However, at the concentrations found in the plasma samples (1.8 mg/ml EDTA and 3.2 mg/ml citrate), POP activity is practically unaffected by EDTA while citrate produces an inhibition of nearly 50% (Fig. 1C). Accordingly, only EDTA-containing plasma was used for further experiments.

### POP activity is altered in remitting-relapsing multiple sclerosis and it is related to disease disability status

**Figure 2:**
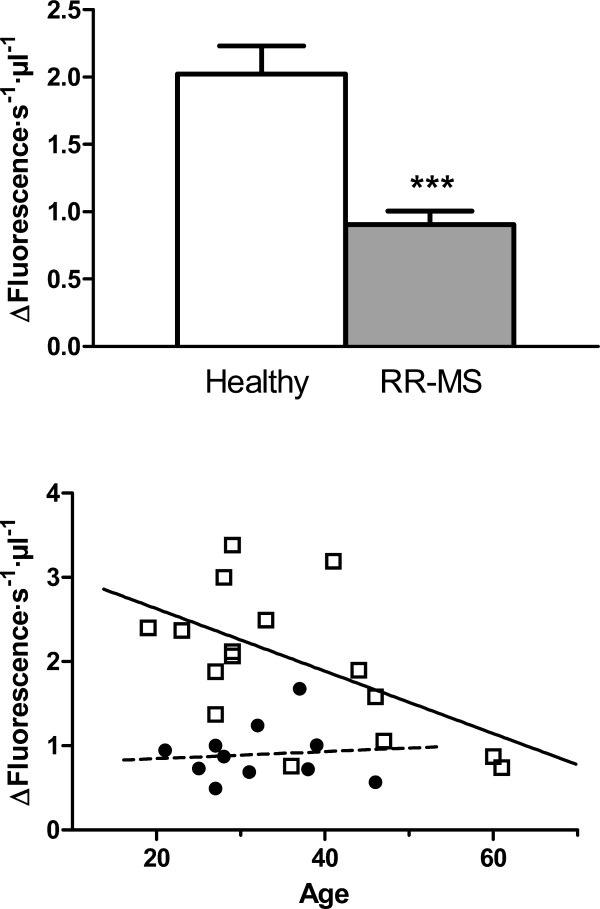
**Prolyl oligopeptidase activity in plasma and the variation with age of healthy subject and RR-MS patients**. POP activity in plasmas from healthy controls (white bar, n = 17) and RR-MS patients (gray bar, n = 11) (A.) ***, *p*-value = 0.0076 t-student unpaired test. B. Relationship between POP activity and subject age for both healthy controls and RR-MS patients. Correlation for healthy controls, n = 17, Pearson r = -0.5153, *p*-value (two-tailed) = 0.03, R-square = 0.2656.

We measured POP activity in plasma of RR-MS patients and compared this with the activity in control samples (Fig. 2). Strikingly, we observed an important decrease in POP activity in RR-MS plasmas relative to controls of about 60% (*p*-value < 0,0005; Fig. 2A). Because MS is a degenerative disease, we considered it relevant to analyze changes in POP activity as a function of patient age and disability status. In Fig. 2B we show the POP activity data from RR-MS patients and controls plotted against age. While there is a clear inverse correlation of POP activity with age for healthy subjects (slope -0.037 ± 0.01; Pearson r = 0,515, *p*-value < 0.05), in RR-MS patients activity remains at medium-low to low level in all ages. It is clear that in the disease state POP activity is already low in younger subjects and no important changes are observed with age.

**Figure 3:**
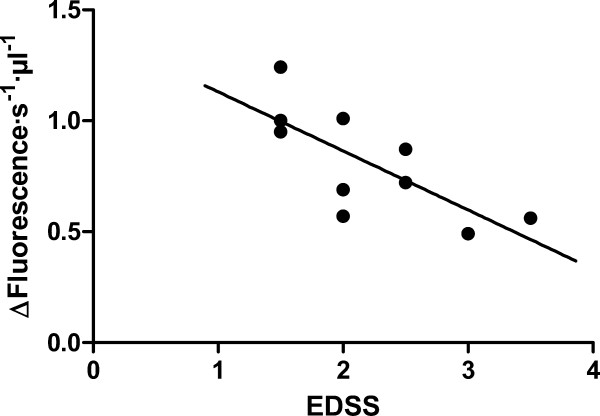
**Prolyl oligopeptidase level and MS disability status**. Correlation between plasma POP activity with the EDSS score from RR-MS patients; n = 10, Pearson r = -0.74, *p*-value (two-tailed) = 0.0144, R-square = 0.5476.

Blood samples analysed were collected from a heterogeneous patient population in terms of disability progression. These disability symptoms ranged from the most benign and slightly disabling, for some patients, to the most aggressive and highly disabling for others. This led us to analyse if changes in POP activity were also related to degree of disability. The Fig. 3 shows POP activity in RR-MS patients related to EDSS score. We clearly found that plasma POP activity decreases with increased disability status (Fig. 2, slope -0.27 ± 0.08; Pearson r = 0.74; *p*-value < 0.05).

### POP endogenous inhibitor levels in plasma are increased in RR-MS patients

**Figure 4:**
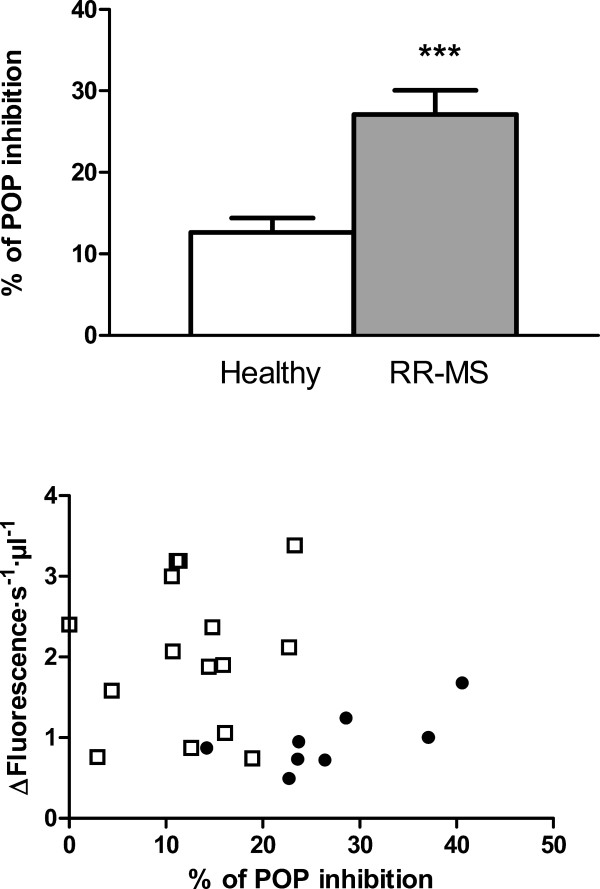
**Levels of endogenous prolyl oligopeptidase inhibitor, and its relation with prolyl oligopeptidase activity, in plasma from healthy and RR-MS patients**. A. Inhibitory effect of plasma (25 μl) from healthy controls (white bar, n = 17) and RR-MS patients (gray bar, n = 11) on the activity of pure POP (basal activity 100 nmol/min/mg of POP), ***, p-value = 0.0002. B. Relationship between plasma POP activity, and endogenous POP inhibitor content, expressed as % of inhibition of pure POP (data from part A.) in healthy controls (n = 17) and RR-MS patients (n = 11). No statistically significant correlation was found.

An endogenous POP inhibitor has been reported before [33] and has been identified as a peptide [34]. This peptide has been proposed as a modulator of POP activity. To verify whether the decrease in POP activity in diseased plasma is due to the action of this or of some hitherto unknown inhibitory factor present in plasma of MS patients, we measured the effect of whole plasma on the turnover of in vitro purified human recombinant POP. As is shown in Fig. 4A, healthy plasma induced a 12% (±2%) inhibition of POP activity while, under the same conditions, diseased plasma doubled this effect, inhibiting the peptidase activity 28% (±3) (*p*-value < 0.0005, healthy vs. diseased). If this increase of inhibitory factor in RR-MS were responsible for the lower POP activity found in MS patients, it might be expected that a correlation would exist between the increase of inhibitory factor and the decrease of activity. When comparing inhibitory factor levels against POP activity in plasma we found no correlation, neither for RR-MS patients nor for healthy subjects (Fig. 4B). Furthermore, we also found no correlation between endogenous inhibitor levels and patient age (not shown).

### Lower POP levels in RR-MS are due to reversible POP oxidation

**Figure 5:**
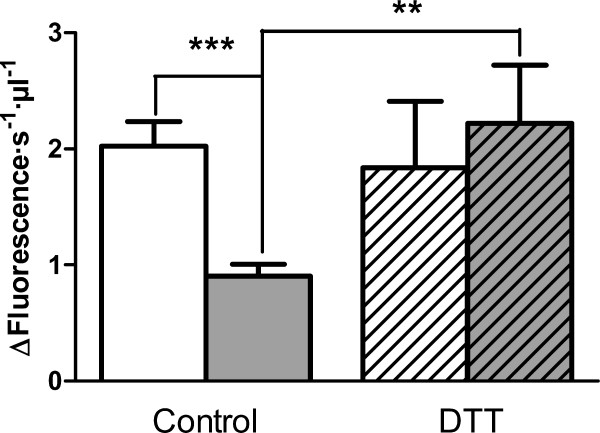
**Effect of DTT on prolyl oligopeptidase levels in plasma from healthy and RR-MS patients**. POP activity in plasma from healthy controls (white bar) and RR-MS patients (gray bar) after plasma pre-incubation in the absence, or presence (diagonal pattern), of DTT (5 mM). n = 10, *p-*values, **, 0.0024, ***, 0.0004.

The increase of POP endogenous inhibitor was not sufficient to explain the substantial decrease in POP activity found in plasma from RR-MS patients. It has been reported that POP is sensitive to redox conditions and is inactivated by oxidants [35,36]. On the other hand, it has been demonstrated that reactive oxygen species (ROS) are substantially increased in MS [37-40]. Hence, we tested the effect of DTT (5 mM) on plasma POP activity. For this purpose, we repeated the POP assay for all samples but after a 5-min pre-incubation with 5 mM DTT. We found that DTT had no effect on POP activity in control samples. However, after pre-incubation with DTT, plasma from diseased patients showed increased POP activity comparable to control levels (*p*-value < 0.005) (Fig. 5).

## Discussion

Many aspects of MS aetiology and pathophysiology are unresolved, but there is increasing evidence that the relapsing-remitting phase and the progressive phase of the disease are caused by two distinct mechanisms. Focal inflammation is believed to be the cause of relapses, whereas diffuse axonal degeneration appears to be the main contributor to progression [41]. The accumulation of disability in MS is thus caused by neuronal damage at inflammatory foci and by diffuse axonal degeneration. Current treatments of MS can reduce the number of relapses but are ineffective in the progressive phase.

POP is a peptidase which cleaves peptides of less than 30 amino acids from the C-side of an internal proline. Since POP is the only proline-specific endopeptidase in humans, and since several proline-containing bioactive peptides are shorter that 30 amino acids, POP has been suggested to specifically modulate the levels of these peptides [1]. Circulating POP levels have been found to be modified in psychiatric [42-47] and mood disorders [31], and these facts have suggested a relationship between these conditions and disturbances in bioactive peptide levels. On the other hand, POP has been found to be related to inflammatory processes through thymosin [23] or collagen degradation [16]. Furthermore, POP has been found to be increased in inflamed tissues [19], and has been found to be secreted from microglia-like cells upon activation [20]. Reactive microglia play an important role in a number of neurodegenerative diseases, including MS [21]. POP has also been reported to interact with cytoskeletal elements with relation to secretion in neural cells [48], suggesting a role in axonal transport. Accordingly, we here hypothesise that POP activity is modified in plasma from MS patients.

In summary, we have found in this study that levels of POP activity in plasma from RR-MS patients are substantially reduced compared with healthy controls (Fig. 2A). Analysing this data, we also find that there is a significant decrease of POP activity with increasing age in healthy subjects. However, this age-associated pattern is not observed for RR-MS patients, where the activity was already substantially lower at early ages (Fig. 2B). Furthermore, we find that the decrease observed in POP activity in diseased plasma is more pronounced in cases with more severe symptoms (Fig. 3). We suggest that the decline in POP activity is related to the molecular mechanism of the disease, and to its severity. In trying to understand the reasons for the low levels of POP which we found to prevail in RR-MS plasma, we analysed plasma looking for inhibitory factors. We found that plasma contained a POP endogenous inhibitor. We observed that levels of this inhibitor are substantially increased in RR-MS, probably partially explaining the low activities found in the disease (Fig 4A). However, we did not find any relationship between the plasma content of this endogenous inhibitor, and the low POP activity observed in diseased plasma (Fig. 4B), which suggests the existence of other factor(s) contributing to this decline. Measures of oxidative stress in blood and cerebrospinal fluid (CSF) of patients with MS have consistently found increases in a number of studies [49,50]. POP is sensitive to the oxidative environment, and it has been reported that oxidation of specific cysteines in this enzyme arrest activity [35,36]. We observed that addition of DTT to plasma from RR-MS patients increases POP to control levels (Fig. 5). This suggests that the higher degree of oxidative stress prevailing in MS inactivates POP.

## Conclusions

We report here that RR-MS patients show decreased circulating POP activity, and that this reduction might be correlated with the higher oxidative environment of plasma of these patients. Additionally, our data indicate that the amount of plasma endogenous POP inhibitor is augmented in this disease, but that this factor is only partially responsible for the decreased POP activities found. We suggest that low amounts of POP activity in MS plasma are contributing to symptoms of the disease. Remarkably, we also found that POP activity is decreased in old age to levels comparable to those found in MS.

On the other hand, the increases observed in levels of POP inhibitory factor in diseased plasma is probably not correlated with increased oxidant levels, since there was no influence of DTT on inhibitory activity in plasma. The relevance of this endogenous inhibitor increase is still not known, and further experimentation is required to answer this question.

Our study is the first to relate POP to MS and indicates that most probably there is a change in peptidase homeostasis in the disease. Further work is being conducted in order to define the proteolytic cascades and the peptides involved in the changes observed in MS. This would contribute to our understanding of disease mechanisms and its symptomatology. According to the results reported here, it can be suggested that levels of plasma POP, and of POP inhibitory factor, could be used as novel tools for early MS diagnosis and also as an indicator of disease severity.

## Competing interests

The authors declare that they have no competing interests.

## Authors' contributions

MB, JAGH and JTL designed the study. MB and JAGH supervised data collection. FCF and BCE supervised neurological examination, and blood sample collection. JTL was responsible for acquisition of data, statistical analysis, and wrote the manuscript. All authors participated in the interpretation of the data, the critical revision of the manuscript, and given approval for the final version.
